# A New Variant of Posterior Canal Benign Paroxysmal Positional Vertigo: A Nonampullary or Common Crus Canalolithiasis

**DOI:** 10.1155/2015/816081

**Published:** 2015-05-31

**Authors:** Sertac Yetiser

**Affiliations:** Department of ORL, Anadolu Medical Center, 41400 Kocaeli, Turkey

## Abstract

Clockwise or counterclockwise, rotational, upbeating nystagmus is seen in patients with posterior canal benign paroxysmal positional vertigo during left or right head-hanging test, respectively. Rotating of nystagmus in opposite direction to the ear tested or even reversal of initial positioning rotational nystagmus is not usual and has never been reported before. We propose a new variant of posterior canal benign paroxysmal positional vertigo due to unusual behavior and location of the otoliths inside the membranous labyrinth. Unexpected rotational direction may lead to confusion about the site. The examiner should be aware of this abnormal or atypical variant of posterior canal benign paroxysmal positional vertigo.

## 1. Introduction

Freely floating otoliths inside the canal or those adhering to the cupula can provoke nystagmus and vestibular disturbance during sudden head motion in patients with benign paroxysmal positional vertigo (BPPV). Diagnosis of posterior canal benign paroxysmal positional vertigo (PC-BPPV) is based on a transient upbeating clockwise or counterclockwise rotational nystagmus in the presence of latency, adaptation, and habilitation associated with a brief and intense sense of vertigo during head-hanging maneuver. Very few studies have been published about simultaneous and spontaneous reversal of peripheral positioning nystagmus during provocative head movements in patients with BPPV. Those are related to canalolithiasis of the lateral canal and posterior canal occurrence has never been reported before [1–3].

Rotating of nystagmus in opposite direction to the ear tested or even spontaneous reversal of initial positioning rotational nystagmus is not usual. We propose a new variant of posterior canal benign paroxysmal positional vertigo due to unusual behavior and location of the otoliths inside the membranous labyrinth. Possible underlying mechanisms have been discussed.

## 2. Case Presentation

**Figure 1:**
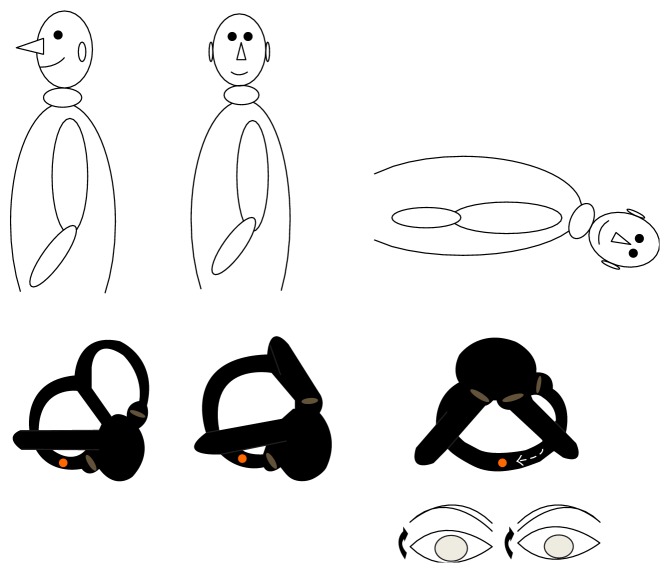
Schematic view of rotatory, torsional, and upbeating nystagmus (clockwise) during head-hanging position in a patient with common left-sided PC-BPPV (dark arrow indicates the direction of the rotation).

**Figure 2:**
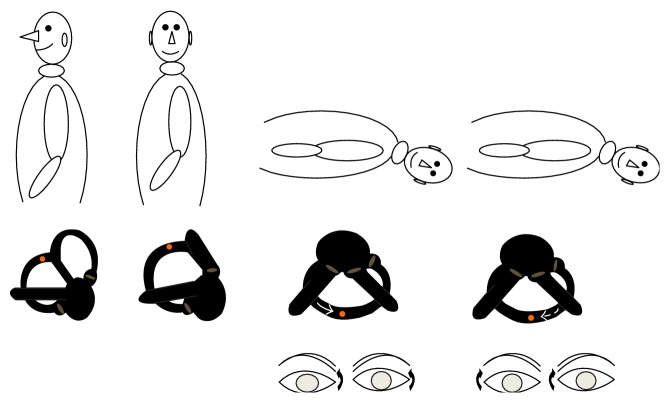
The mechanism of spontaneous inversion of nystagmus in a patient with left-sided PC-BPPV (dark arrow indicates the direction of the rotation).

A 38-year-old woman with a medical story of head induced vertigo for less than two weeks was admitted to the outpatient clinic. She had no evident associated problem and could be defined as an “idiopathic” case. A verbal consent and a signed informed consent were obtained from the patient. The procedures were in accordance with the ethical standards of the Declaration of Helsinki and of the institutional review board. Diagnosis of PC-BPPV was based on medical history and the presence of upbeating rotational nystagmus during head-hanging maneuver when the affected ear is down. However, there was no nystagmus on the right side. But, on the left side, counterclockwise, rotational, torsional, and upbeating nystagmus was seen initially which was later followed by clockwise rotational nystagmus while keeping the head at the hanging position which confirmed a left-sided PC-BPPV. The patient was also subjected to head-roll and straight head-hanging maneuvers which were not remarkable. Inversion of the nystagmus was documented when the patient got back to sitting position. Videonystagmography (Micromed, Inc., USA) recording confirmed a new variant of PC-BPPV (*video-link*) (see Supplementary Material available online at http://dx.doi.org/10.1155/2015/816081). Latency was very brief (5 seconds). First phase of nystagmus appeared immediately after bringing the patient's head to head-hanging position and lasted 15 seconds followed by a “silent phase” for another 10 seconds as seen on video. Then, a second phase of nystagmus appeared lasting for 40 seconds. Whole event happened in 80 seconds. The intensity of the first-phase nystagmus was stronger than that of second phase. Maximal slow phase velocity was calculated as 6 and 3 degrees/sec for the first phase and second phase of nystagmus, respectively. However, the second-phase nystagmus lasted longer. The patient was completely cured after one single Epley maneuver for the left side. Schematic view of rotatory, torsional, and upbeating nystagmus (clockwise) during head-hanging position in patients with a common left-sided PC-BPPV is seen in Figure 1. The mechanism of spontaneous inversion of nystagmus in an atypical PC-BPPV is demonstrated in Figure 2.

## 3. Discussion

Counterclockwise, upbeating, rotational nystagmus when the head is at left hanging position as seen in this case initially is pretty much related to the abnormal location of the otoliths in the long arm of the canal, close to the common crus. Otoliths are always located close to the cupula in the posterior canal most probably due to gravitational effect which leads to accumulation of debris always in the lower part. Therefore, when the head is briefly brought to head-hanging position, free-floating otoliths close to the ampulla cause ampullofugal deflection (pulling cupula) and an excitatory clockwise upbeating rotational nystagmus (Figure 1). It is always clockwise since stimulation of the ipsilateral side activates ipsilateral superior oblique and contralateral inferior rectus muscles.

However, if the otoliths are located in the long arm of the posterior canal close to the common crus it may result in ampullopetal flow initially. When the head is brought to left hanging position, debris will fall under the influence of gravity creating an endolymphatic flow toward the ampulla which will cause inhibition of the ipsilateral side and stimulation of the contralateral side which also leads to activation of contralateral superior oblique and ipsilateral inferior rectus muscles. Their contraction will result in torsional counterclockwise upbeating nystagmus as seen in this case. Eventually, the cupula deflection will end when the particles reach their lowest position as the head is kept at hanging position. Then, a burst of endolymphatic reflux in the opposite direction dragging the clot away from the cupula will result in clockwise upbeating nystagmus when maintaining the head rotation to the pathological left side.

Spontaneous reversal of nystagmus in this case indicates that at least ampullopetal as well as ampullofugal flows can occur during single head movement. Reversal of initial positioning nystagmus in benign paroxysmal positional vertigo during maintaining the head position is quite unusual and an interesting finding. This phenomenon is defined as the appearance of a reverse nystagmus in the opposite direction after cessation of the original provoked positional nystagmus during head movement. Translation of torsional counterclockwise nystagmus on the left ear's head-hanging position to the true clockwise nystagmus is quite interesting. Reversal of positioning geotropic nystagmus to apogeotropic nystagmus in cases with lateral canal BPPV has been attributed to short-term adaptation of the vestibule-ocular reflex [2–4]. However, it is unlikely to explain this rare condition with this theory. We assume that the reversal of nystagmus in this case is due to second-phase endolymphatic flow due to reversal of clot movement. First-phase nystagmus was more intense than that of second phase. But the duration of second phase was longer. This could be due to influence of the different forces affecting the otolith movement. First-phase force is gravitational, instantaneous, fast, and strong with head movement and second-phase force is long-lasting, slow, as the otoliths being dragged along. But of course, both were weaker than usual PC-BPPV nystagmus due to dispersal of the otoliths.

Superior canal benign paroxysmal positional vertigo (SC-BPPV) and nonampullary PC-BPPV are quite rare due to basically anatomic considerations since the superior canal is higher than both posterior and lateral canals and posterior arm of superior canal descends directly into the common crus which explains continuous self-clearing of the otoliths from the canal. Otoliths found at the superior canal can stimulate posterior canal or SC-BPPV can somehow turn into PC-BPPV. In 1995, Agus described a “reversed” PC-BPPV with a downbeating positional nystagmus characterized by torsional components clockwise for the right and counterclockwise for the left head-hanging positioning [5]. Cambi et al. have followed patients with positional downbeating nystagmus and indicated conversion of some of them to PC-BPPV [6]. Vannucchi et al. have reported a group of 6 subjects among 45 patients with torsional downbeating nystagmus clockwise for the right and counterclockwise for the left head-hanging position. These patients had typical PC-BPPV of the opposite side a few days later during second visit. They called this new form of BPPV “apogeotropic PC-BPPV” [7]. All patients had initial diagnosis of SC-BPPV and were treated as having SC-BPPV.

The presented geotropic form of PC-BPPV is different from all previously reported cases due to its unique and evidence-based characteristics. Typical nystagmus due to posterior canal excitation should be expected in a vertical-torsional pattern having the linear component of its fast phase directed upward. Reversal of rotational axis in this case could be explained by the presence of first ampullopetal and then ampullofugal flow of the endolymph during single head movement. Otoliths located at the nonampullary side of the posterior canal that could lead to a downbeating nystagmus need more clarification since there is always a component of previous superior canal involvement in the reported cases. Besides, we do not prefer to use the term “apogeotropic” for patients with downbeating nystagmus which may lead to confusion in the nomenclature since apogeotropic nystagmus of the lateral canal describes the otoliths located close to the cupula and apogeotropic nystagmus of the posterior canal describes the otoliths located away from the cupula. However, otoliths located away from the cupula in the posterior canal may cause geotropic nystagmus as seen in this case. The term of nonampullary PC-BPPV or common crus PC-BPPV is more suitable to describe this atypical geotropic posterior canal canalolithiasis with reversal of rotational axis. However, this is an evolving subject. The location, type of dispersion, or the nature of debris could be different as well. As new interesting cases detected this issue will be clearer and a more appropriate classification of PC-BPPV is going to appear.

## 4. Conclusion

Reversal of spontaneous nystagmus is a rare condition and is basically due to endolymphatic flow and clot movement in the opposite direction away from the cupula which is characteristic feature of canalolithiasis. Our data indicate that the reversal of positioning of the nystagmus in this patient with PC-BPPV is related to unusual location of the otoliths in the long arm or the nonampullary end of the posterior canal. Atypical counterclockwise torsional upbeating nystagmus on the left head-hanging position is followed by true clockwise nystagmus by inversion of the direction of clot movement due to spontaneous reflux of the endolymph. Unexpected rotational direction may lead to confusion about the site. The examiner should be aware of this abnormal or atypical variant of PC-BPPV.

## Supplementary Material

Spontaneous reversal of axis of the up-beating nystagmus from counter clockwise to clockwise rotation at left head-hanging position.

## Abbreviations

BPPV: Benign paroxysmal positional vertigo
PC-BPPV: Posterior canal benign paroxysmal positional vertigo
SC-BPPV: Superior canal benign paroxysmal positional vertigo.
